# Population Development of *Tuta absoluta* (Meyrick) (Lepidoptera: Gelechiidae) under Simulated UK Glasshouse Conditions

**DOI:** 10.3390/insects4020185

**Published:** 2013-05-15

**Authors:** Andrew G.S. Cuthbertson, James J. Mathers, Lisa F. Blackburn, Anastasia Korycinska, Weiqi Luo, Robert J. Jacobson, Phil Northing

**Affiliations:** 1 The Food and Environment Research Agency, Sand Hutton, York, YO41 1LZ, UK; E-Mails: james.mathers@fera.gsi.gov.uk (J.J.M.); lisa.blackburn@fera.gsi.gov.uk (L.F.B.); anastasia.korycinska@fera.gsi.gov.uk (A.K.); weiqi.luo@fera.gsi.gov.uk (W.L.); phil.northing@fera.gsi.gov.uk (P.N.); 2 Rob Jacobson Consultancy Ltd., 5 Milnthorpe Garth, Bramham, LS23 6TH, UK; E-Mail: rob.jacobson@tiscali.co.uk

**Keywords:** integrated pest management, lifespan, population development, tomato

## Abstract

Tomato leafminer *Tuta absoluta* (Meyrick) is a major pest of tomato plants in South America. It was first recorded in the UK in 2009 where it has been subjected to eradication policies. The current work outlines *T. absoluta* development under various UK glasshouse temperatures. The optimum temperature for *Tuta* development ranged from 19–23 °C. At 19 °C, there was 52% survival of *T. absoluta* from egg to adult. As temperature increased (23 °C and above) development time of the moth would appear to decrease. Population development ceases between 7 and 10 °C. Only 17% of eggs hatched at 10 °C but no larvae developed through to adult moths. No eggs hatched when maintained at 7 °C. Under laboratory conditions the total lifespan of the moth was longest (72 days) at 13 °C and shortest (35 days) at both 23 and 25 °C. Development from egg to adult took 58 days at 13 °C; 37 days at 19 °C and 23 days at 25 °C. High mortality of larvae occurred under all temperatures tested. First instar larvae were exposed on the leaf surface for approximately 82 minutes before fully tunnelling into the leaf. Adult longevity was longest at 10 °C with moths living for 40 days and shortest at 19 °C where they survived for 16 days. Generally more males than females were produced. The potential of *Tuta absoluta* to establish populations within UK protected horticulture is discussed.

## 1. Introduction

**Figure 1:**
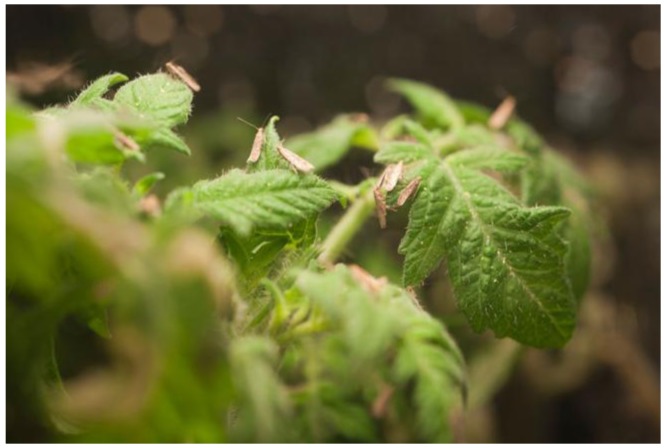
Adult *Tuta absoluta* on tomato foliage (UK Crown copyright^©^).

The introduction of non-indigenous pests and diseases into a country can impact on both forestry and horticultural industries [1,2], where crop damage and even complete loss may occur. Environmental damage has also been recorded following establishment of non-indigenous organisms [3]. The tomato leafminer *Tuta absoluta* (Meyrick) (Lepidoptera: Gelechiidae) (Figure 1) is one such pest, originating from South America, that devastates tomato plants [4,5,6,7,8]. Within Europe it was initially detected in the Iberian Peninsula in 2006 [9]. Since then it has rapidly moved across the Mediterranean area and has been detected in France, Italy and the United Kingdom (UK) [10,11]. In 2009 there were 11 outbreaks of *T. absoluta* in the UK followed by 15 in 2010, 8 in 2011 and 7 in 2012 [12].

Studies regarding the basic biology and population development of *T. absoluta* are relatively few and are mainly concentrated in South American countries where the environmental conditions are very favourable for the life-cycle of the pest [5]. Females of *T. absoluta* deposit their eggs on leaves, stems and petioles. The four larval instars usually feed and develop in leaf mines in the inside of the leaf between the upper and lower epidermis but may also be found inside fruit and stems. The pupae are principally found in the ground or on the fabric of the greenhouse but may also occur on the tomato plant. Damage produced by this pest is focused on the larval galleries made on the leaves, the terminal buds, the flowers and the fruits of the tomato crops [13,14]. It has been reported that this pest can cause reductions in crop yield of up to 90% [15]. The current study investigates the population development of *T. absoluta* under environmental conditions associated with UK tomato glasshouses. Also the time period that first instar larvae remain on the leaf surface before burrowing into the leaf surface was determined. Knowing this information can enable the correct timing of application of biological control agents against the pest.

## 2. Experimental Section

### 2.1. Source of Tuta absoluta and Maintenance of Culture Stock

Specimens of *T. absoluta* were obtained from an outbreak within a commercial glasshouse in Portugal in 2010. They were imported into the UK under license (as required for all other non-indigenous insects [16]) and maintained within the Plant Health Insect Quarantine Unit at Fera, York. Cultures were initiated and maintained within sealed Perspex cages (60 cm × 60 cm × 80 cm) on tomato plants (*Lycopersicon esculentum* c.v. Moneymaker) similar to those used for other non-indigenous insects [17] at 20 °C, 65% r.h. and a 16:8 h L:D regime.

### 2.2. Population Development under Various Temperatures

The rate of development of *T. absoluta* was investigated under the following constant temperatures: 7, 10, 13, 19, 23 and 25 °C. Development was also investigated under the fluctuating temperature of 23 °C for 16 h (day) and 18 °C for 8 h (night). The relative humidity for all trials was 65%. This would give approximately an average temperature of around 21 °C, similar to conditions in UK glasshouses. Ten newly laid (all within 24 h of being laid) eggs were placed on an individual tomato leaf the end of which was then maintained in cotton wool in a microcentrifuge tube with water. The leaf was then placed in a plastic box, lined with moist tissue, with a mesh lid to allow air movement and maintained in a controlled environment (CE) cabinet at the desired temperature. The tissue paper was moistened daily. The experiment was replicated ten times giving 100 eggs per trial (each temperature) in total. Once eggs had hatched and larvae had entered the leaves, each leaf was placed into an individual Petri dish (15 cm diameter) with fresh leaves added as required. This was then sealed with Parafilm^®^ and placed in the appropriate CE cabinet. This allowed adult moths emerging from individual leaves to be counted. These moths had no food supply. The following data was recorded for each temperature: time taken for eggs to hatch, percentage egg hatch, time taken to develop from larvae to adult, percentage adult emergence, adult longevity without food, sex of adult moths emerged.

### 2.3. Sexing Tuta absoluta Moths

The moths were sexed by examining their genitalia through dissection. The hindwing frenulum was also checked. In general male moths have genitalia with 2 valves that can be seen as a slit on the ventral side of the abdomen at the posterior end. The frenulum on the anterior edge of the hind wing consists of a single thick bristle. The abdomen is usually slender, often parallel-sided. Female abdomens will taper to a point posteriorly, possibly with a visible ovipositor, but always with no central slit. The frenulum consists of at least two bristles. The abdomen may look more swollen, and is less likely to be parallel-sided.

### 2.4. Larval “Wandering” Time on Leaf Surface

To assess and determine the time first instar larvae spend on the leaf surface after hatching and before they burrow into the leaf surface to continue their lifecycle and development, eggs were brought through to an almost hatching stage at 19 °C (determined by visible head capsules through the egg chorion/shell). An individual larva as it hatched was observed manually under a light microscope while others were placed under a time lapse camera microscope with the aim of recording larval behaviour. The hatching eggs were observed and the time the larvae spent on the leaf surface following hatching until full submergence into the leaf structure was recorded. This procedure was repeated on three separate occasions for individual larvae, giving three records in total.

### 2.5. Adult Moth Longevity When Supplied with Food Source

The longevity of adult moths at the various temperatures stated above was determined by taking a newly emerged (less than 24 h old) adult moth and placing it within a 0.5 litre plastic container containing a honey/water solution as a readily available food source. The experiment was replicated 15 times for each temperature tested. Mortality of moths was assessed daily.

### 2.6. Data Analysis

Data was analysed where appropriate. Assuming normality and constant variance, analysis of variance (ANOVA) was used to test any significant difference between different treatments (temperature). Pairwise comparison (with FDR multiple correction) was used to quantify the differences.

## 3. Results

### 3.1. Population Development under Various Temperatures

**Figure 2:**
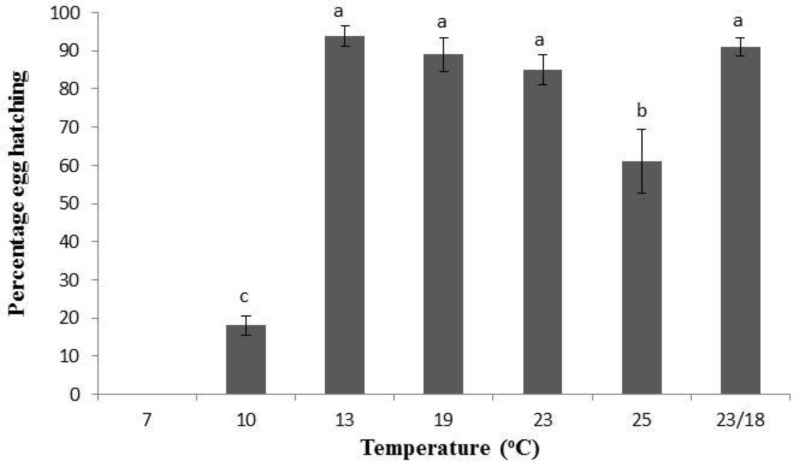
Percentage of *Tuta absoluta* eggs hatching at various temperatures (°C). Different letters denotes significant differences (*p* < 0.05).

No eggs hatched at 7 °C. At 10 °C only 17% of eggs hatched (Figure 2), significantly less than all the higher temperatures (*p* < 0.001). Eggs maintained at 13 °C had the highest percentage hatch with 92% hatching though this was not significantly more than at 19 °C (*p* = 0.47). Temperatures between 13 °C and 23 °C all had hatch rates above 80% and the fluctuating temperature, with the daily average of 21 °C, fitted into this overall pattern. There was a significant decline in egg hatch above 23 °C (*p* < 0.05).

**Figure 3:**
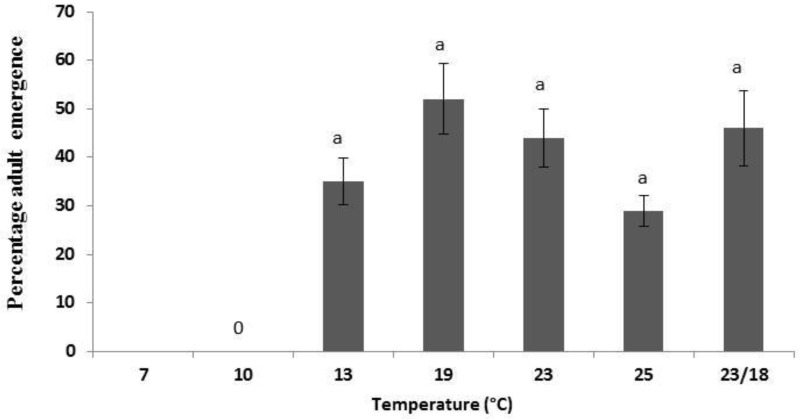
Percentage emergence of *Tuta absoluta* adults at various temperatures. Different letters denotes significant differences (*p* < 0.05).

The highest adult emergence occurred at 19 °C (*p* = 0.42) with 52% of eggs surviving through to adult moths (Figure 3). Even though eggs hatched at 10 °C only 3% of eggs reached the pupal stage, however these died and therefore no adults emerged. Adult emergence rates also declined, after peaking at 19 °C, with increasing temperature (Figure 3). As with egg hatch, the fluctuating temperature, with the daily average of 21 °C, fitted into the overall pattern. There was larval mortality rates of greater than 50% (assuming all eggs that hatched were viable), for example, at 13 °C there was 92% egg hatch but only 40% adult moth emergence.

**Figure 4:**
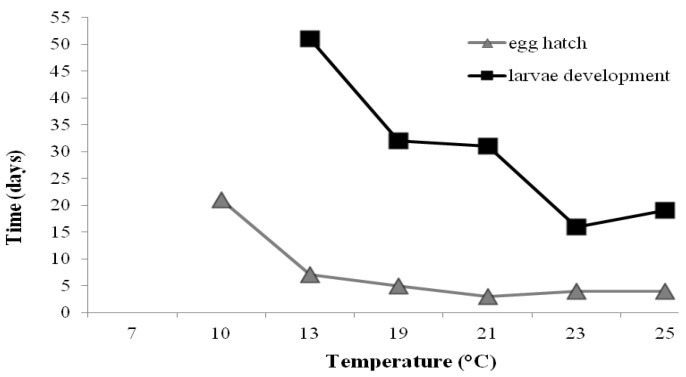
Time-span for *Tuta absoluta* egg and larval development at various temperatures.

More male moths were observed to develop in the trials than female, though results were not significantly different (*p* = 0.67) (Table 1). Eggs of *T. absoluta* took longest to hatch at 10 °C (21 days) and least time (3 days) with the fluctuating temperature of 23/18 °C (average 21 °C) (Table 2; Figure 4). However at 10 °C no adult moths developed through. Larvae remained developing in the leaves longest at 13 °C (51 days) (Table 2) (*p* < 0.05) and consequently had the longest life-span of 72 days. The adults all lived for between 10–15 days with no food source available (Table 2).

**Table 1:** Number of male and female adult *Tuta absoluta* emerging at various temperatures. Different letters within columns denotes significant differences (*p* < 0.05).

Temperature (°C)	Male	Female	Total
7	0	0	0
10	0	0	0
13	23 ^a^	17 ^a^	40
19	27 ^a^	25 ^a^	52
23	25 ^a^	19 ^a^	44
25	17 ^a^	12 ^a^	29
23/18	18 ^a^	31 ^a^	49
Total	110	114	214

**Table 2:** Time-span in days of *Tuta absoluta* life-stages developing at the various temperatures tested (no food supplied for adult moths). Different letters within columns denotes significant differences (*p* < 0.05).

Temperature (°C)	Egg hatch	Larvae	Adult	Total life-span
7	-	-	-	-
10	21 ^a^	27 ^a,^*	-	-
13	7 ^b^	51 ^b^	14 ^a^	72
19	5 ^b^	32 ^a^	14 ^a^	52
23	4 ^b^	16 ^a^	15 ^a^	35
25	4 ^b^	19 ^a^	12 ^a^	35
23/18	3 ^b^	31 ^a^	10 ^a^	44

* Did not survive to adult.

### 3.2. Adult Moth Longevity When Supplied with Food Source

**Figure 5:**
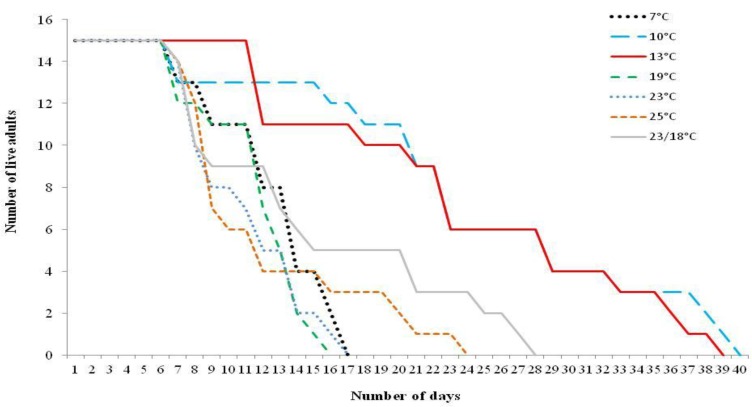
Adult *Tuta absoluta* longevity at various temperatures (°C).

Newly emerged adult *T. absoluta* survived longest at 10 °C (Figure 5). Here the adults lived for 40 days being offered a honey/water solution as a food source. This was significantly longer (*p* < 0.01) than the shorter survival rates noted for 19 °C (16 days) and 7 and 23 °C (both 17 days respectively). 

### 3.3. Larval “Wandering” Time on Leaf Surface

**Figure 6:**
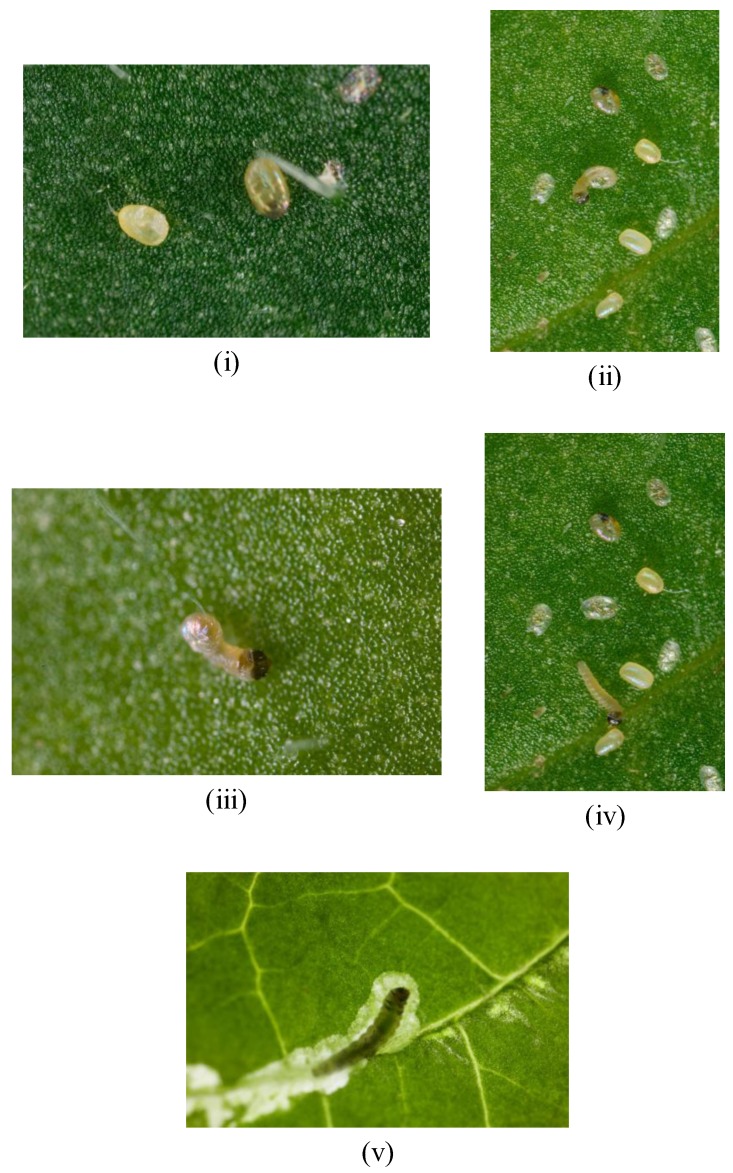
(**i**–**v**). *Tuta absoluta* larvae hatching from egg shell and wandering on leaf surface before tunnelling into leaf (UK Crown copyright^©^).

First instar larvae took approximately 18 minutes to hatch out of the shell once hatching began (Figure 6i–iii). Once hatched, the larvae wandered around the leaf surface for an average of 12 minutes and approximately 15 mm from its egg shell before starting to graze on the leaf surface (Figure 6iv). From this moment onwards the larvae started to burrow under the leaf surface and after a further 70 minutes were totally encased inside the leaf (Figure 6v).

## 4. Discussion

*Tuta absoluta* is well able to develop under temperatures that would commonly be experienced in UK glasshouses. The current work has determined that between 19 and 23 °C is the most favourable temperature for moth development. Temperatures of 10 °C and below proved fatal for moth development. It cannot be confirmed that a population would die out if glasshouse temperatures were lowered to this temperature. However, this could form the basis of a control programme for sterilizing the glasshouse following an outbreak of moths. Eggs did hatch at 10 °C but the lifecycle never completed, several first instar larvae were noted dead on the leaf surface having failed to burrow into the leaf. It is assumed that full mortality was caused in the larval stage. No eggs hatched at all when held at 7 °C, they simply rotted. From this work we can determine that 10 °C is at the lower end of the temperature range for *T. absoluta* development. Temperatures above 23 °C appeared to have a detrimental effect on *T. absoluta* development and life-span. This would suggest that UK glasshouse conditions will be very favourable for this pest to develop compared to hotter (for example Mediterranean) climates. This conclusion fits with a species that originates from the foothills of the Andes in South America. These findings are also consistent with reports from growers in southern Italy who have reported that population growth of *T. absoluta* is greatest in spring/early summer and again in late summer/autumn with a period of respite in mid-summer [18]. However, *T. absoluta* has become a pest in many Mediterranean countries [19], so there would appear to be much variation within its ability to develop under differing temperatures.

In the current study *T. absoluta* showed high natural mortality. During its life cycle the larval stage is known to be the most critical [5]. In the field, it has been suggested that third-instar mortality could be due to the dispersal of the caterpillars as they grow, which would increase their exposure to predators. On the other hand, larvae of first and second instars remain in the leaf where oviposition takes place. When they reach the third instar there is more competition for food and therefore the larvae need to spread over the tomato plant [20]. During the fourth stadium most of the caterpillars are in different leaves, but they feed in the same mine throughout this stadium. High mortality in instars could also be due to the fact that early instars are closer together and therefore more vulnerable to predators in the field. Under laboratory conditions in the current study presence of natural enemies was not a problem. The study therefore determines that *T. absoluta* has naturally high larval mortality. 

High egg mortality is not a rare characteristic among insect species [20]. Poorly protected eggs on the leaf surface are an easy target for predation and parasitism, but the oviposition of large numbers of eggs, many of which may not be viable, may minimise the impact of these mortality factors on the pest population. This may also be an explanation for the low numbers of adult moths developing through in our laboratory experiments compared to the starting number of eggs. Our experiments showed that in general more males than females developed through in all the trials except the fluctuating temperature trial. However, it was basically a 1:1 ratio. This is the reverse of that found by Fernandez and Montagne [21] who observed more females than males in general. 

*Tuta absoluta* is multivoltine and population parameters suggest that it is an “r” selected species [22]. The duration of the developmental cycle greatly depends on environmental conditions, with average development time of 76.3 days at 14 °C, 39.8 days at 19.7 °C and 23.8 days at 27.1 °C [4]. These figures differ from those obtained within the current study. We found total development from egg to adult only took 58 days at 13 °C; 37 days to develop at 19 °C and 23 days at 25 °C. *Tuta absoluta* would have appeared to have developed faster from egg to adult in our studies that the times recorded in Barrientos *et al.* [4] study. Vercher *et al.* [23] stated that they were able to maintain and keep *T. absoluta* larvae alive during several weeks at 4 °C, however, in our study no larvae survived 10 °C when maintained at this temperature with ample fresh tomato foliage supplied. We also had no egg hatch at 7 °C, all simply rotted after being maintained at this temperature for up to 66 days.

Desneux *et al.* [10] cite information from Estay [24] that adult *T. absoluta* lifespan ranges between 10 and 15 days for females and 6–7 days for males. However, it is totally contradicted by the current study. Though the adult *T. absoluta* were not sexed in our longevity study they lived much longer than 15 days. In the life stage development trials undertaken (where adult moths had no food supplied) they lived for up to 15 days at 23 °C. In the longevity trials where adult moths were supplied with a food source of honey/water they survived for 40 days at 10 °C. The physiological age of insects is known to increase at lower temperatures [25]. It is also suspected that the moths are simply not as active at the lower temperatures. They are also probably not mating as frequently or laying as many eggs at lower temperatures.

Egg laying by female moths was not recorded in the current study but is stated by Uchoa-Fernandes *et al.* [26] that females mate only once a day and are able to mate up to six times during their lifespan, with a single mating bout lasting 4–5 h. The most prolific oviposition period is 7 days after first mating, and females lay 76% of their eggs at that time, with a maximum lifetime fecundity of 260 eggs per female.

The primary objective of this study was to provide biological data to underpin control measures for *T. absoluta* that could be incorporated into the existing integrated pest management (IPM) programme for UK tomato crops. Current IPM programmes have been developed over 30 years and are highly advanced. It includes multiple control measures simultaneously employed against over 10 individual species of pests [27]. If it becomes necessary to resort to non-specific insecticides against *T. absoluta*, then the whole IPM programme could be disrupted. The first step in developing an IPM compatible strategy against a new pest is to identify weak points in its life cycle which could be exploited by novel control measures. For *T. absoluta*, the weak points would appear to be the egg and the first instar larva before it tunnels into the plant. The eggs are robust but are laid in exposed positions on the surface of leaves, stems and petioles where they are vulnerable to attack by parasitoids and predators. Kabiri *et al.* [28] and Cabello *et al.* [29,30] describe successful trials in which large numbers (over 1 million/ha/week) of the egg parasitoids, *Trichogramma achaeae* (Hymenoptera: Trichogrammatidae), were released prophylactically, starting as soon as crops were planted. However, many IPM practitioners have questioned the cost-benefit of these very high release rates. Molla *et al.* [31] and Urbaneja *et al.* [32] reported that the predatory bugs, *Nesidiocoris tenuis* and *Macrolophus pygmaeus* (Hemiptera: Miridae) prey on *T. absoluta* eggs under laboratory conditions. Similarly, Cabello *et al.* [33] demonstrated that the damsel bug, *Nabis pseudoferus* (Hemiptera: Nabidae) will feed on *T. absoluta* eggs. Nonetheless, the very large numbers of eggs laid by each *T. absoluta* female [5] indicate that extreme levels of egg predation would be required to prevent population growth.

Between hatching from the egg and tunnelling into the plant tissue, the first instar *T. absoluta* larva could be vulnerable to numerous chemical insecticides, biopesticides and predators. However, once inside the plant, control options are greatly reduced. Prior to this study, unsubstantiated reports from workers based in the Mediterranean region suggested that first instar *T. absoluta* larvae spend up to 36 h on the leaf surface before entering the plant tissue [18]; *i.e.*, up to 10% of the larval development period at 23 °C. The present study has determined that the first instar is only exposed on the leaf surface for approximately 82 minutes, which is less than 0.005% of the larval development period at the same temperature. The biopesticide, *Bacillus thuringiensis*, has been shown to kill larvae of *T. absoluta* under experimental conditions [34] but its success is dependent upon contact with, or ingestion by, the pest. In theory, the larvae should only collect a lethal dose when outside the mine and so the most vulnerable life cycle stage should be the free living phase of the first instar. In practice, later instars may also be vulnerable if they move between leaves. Nonetheless, the product would have to be applied at frequent (perhaps less than weekly) intervals to have an impact on the *T. absoluta* population growth. The same would apply to any chemical insecticides which are dependent on contact action on the leaf surface unless they are very persistent. However, the longer harvest intervals of persistent insecticides usually preclude their use on tomato crops because fruit is harvested every 2–3 days.

Scrutiny of the pest’s life cycle indicates that control measures must target *T. absoluta* larvae within the tomato leaves. An effective and acceptable chemical insecticide must therefore penetrate the leaf, have a harvest interval of less than three days and be compatible with all the biological control agents employed in current tomato IPM programmes [35,36]. Very few insecticidal products meet all these criteria. However, several biological control agents can attack *T. absoluta* larvae within leaves in a commercial tomato crop. These include the predatory bugs, *N. tenuis* and *M. pygmaeus* [11,31] which probe into the leaf to find the caterpillars. Larval parasitoids such as *Necremnus artynes* (Hymenoptera: Chalcidoidea), which paralyse the caterpillar before laying an egg adjacent to it, may also have potential in commercial crops [37,38]. Jacobson and Martin [39] describe how high volume foliar sprays of the entomopathogenic nematode, *Steinernema feltiae*, could also make an important contribution to the overall IPM programme by slowing down the population growth of *T. absoluta* while the primary biological control agents (*Nesidiocoris tenuis* and/or *Macrolophus* spp.) become established. The addition of food supplements can also significantly impact on *M. pygmaeus* population development [40]. Their findings were broadly consistent with those of Batella-Carella *et al.* [41]. This technique could be particularly important in organic tomato crops where there are very few effective alternatives.

## 5. Conclusion

*Tuta absoluta* has huge potential to establish populations within the UK protected horticulture industry [42]. Understanding the population development of the pest under specific conditions will aid in the formation and application of integrated control strategies against the pest.
